# Do You See Me?

**DOI:** 10.4269/ajtmh.19-0438

**Published:** 2019-12

**Authors:** Tara E. Ness, Amanda T. Small

**Affiliations:** 1 Baylor College of Medicine, Houston, Texas;; 2 Texas Children’s Hospital, Houston, Texas

From behind my back I hear the traditional siSwati greeting of “Sawubona,” which translates literally to “Do you see me?” My patient, whom I will call Nompilo, hands me her “green book”—a record of her HIV management over the years. Nompilo is a 19-year-old woman, and just from glancing down at her book, I can see this is going to be a long visit. She has been on antiretroviral therapy for years, and her viral load has continued to creep up, indicating either she has been noncompliant with her medications or the medicines have stopped working. I finally look up and take a chance to respond to her initial greeting.

“Yebo,” I say. “Yes, I see you.”

We are in Eswatini (formerly known as Swaziland), a land-locked country, bordered by Mozambique and South Africa, and one of the last absolute monarchies in the world. It is characterized by diverse landscapes ranging from beautiful mountains to savannas and tropical forests.

Amid the beautiful scenery is an HIV prevalence of 27%, the highest in the world. Nompilo is among a newly emerging population in Eswatini: adolescents and young adults with vertically transmitted HIV. Nompilo was born during a time when mother to child transmission of HIV ranged from 25% to 40%, which included transmission during pregnancy, birth, or breastfeeding, because of the lack of antiretroviral therapies available. The prevention of mother-to-child transmission program did not begin in Eswatini until 2003, and only in the last few years, with development of more effective antiretroviral regimens, has the rate of vertical transmission decreased to 2%.

Nompilo, like other children with HIV, was at high risk of fast disease progression, with a high mortality rate before the widespread use of antiretrovirals that gained momentum in 2006. Now, over a dozen years later, she and her peers are part of a new demographic of people born before the successes of the prevention of mother-to-child transmission program, yet late enough to benefit from antiretroviral treatment in childhood. As a 19-year-old, having HIV poses distinct challenges to an already tumultuous period of life.

When I talk to Nompilo, she reminds me of the other adolescents in my clinic who have shared with me similar stories. They face the typical teenage challenges of fighting for autonomy and independence, social pressures at school, and navigating a new world of drugs, alcohol, and dating. In addition, they must also battle with a lifelong disease linked to serious moral stigma.

Imagine what it feels like having to take a pill every day and hide your chronic illness from your peers for fear of stigma. You worry if your romantic partner will reject you if you disclose your status. When you get off the bus at the clinic to get refills of your medicine, everyone on the bus suddenly realizes you are HIV positive and may shake their head or make a motion like they are avoiding physical contact with you. People may make assumptions about your sexual activity and the way you obtained HIV and you have no opportunity to defend yourself.

Imagine returning home to your family, where none of your younger siblings has HIV and they do not have to make the trip to the clinic. You may wonder why you are the only one of your siblings who has HIV.

You may feel angry and want someone to blame, but your parents passed away from HIV years ago, so you have no one.

You feel ashamed, angry, alone, and the last thing you want to do is pick up a pill each morning and remind yourself that you have HIV.

You skip a pill and feel fine. You skip a few more. On top of the natural immortality complex that some of this age-group feels, HIV can be especially difficult because until your immune system begins to really fail, you do not feel sick. Nompilo’s detectable viral load is not just a number; it represents an ongoing, multifaceted, and deeply emotional conflict in her life.

Being an effective health-care provider for this population is difficult. There is no perfect answer of how to address all of the unique challenges that Nompilo and her peers face. At our site, Nompilo is part of a monthly “Teen Club,” where teenagers from our clinic get together and sing, dance, eat snacks, and talk about their plans for the future. Teen Club is the kind of intervention that provides a community for these teenagers, to lessen the loneliness of living with a chronic disease. Nompilo is also attending a theater camp we are piloting this fall, which we hope will give our young adults an outlet for sharing their story and provide a forum for creative, artistic expression.

We also encourage caregivers to teach their children about HIV early in their life, long before the teenage years. A fantastic book, “Our Little Soldiers,” written by a graduate of our global health residency program, assists parents in disclosing HIV, by framing CD4 cells as “soldiers” that protect their body from germs. Early and full disclosure is difficult to study, but research has shown improved adherence and retention in care with early disclosure of HIV status to children.

Recently, another patient of mine, whom I will call Lindokuhle, returned to the clinic after he had “defaulted” and completely stopped taking his HIV medications. The children at his school had found out his status and he had been devastated by the social challenges he had to face. One of his peers from the clinic’s Teen Club, we will call him Mefika, without prompting from our staff, went to the patient’s house and talked to him. They talked about the challenges they both faced, and Mefika successfully convinced Lindokuhle that he should continue taking his medicine. Lindokuhle was reminded he was not alone.

The situation of adolescents and young adults with HIV is not limited to Eswatini. This is reflected by the fact that the WHO highlighted the need for specific HIV services for adolescents in 2013, encouraging social support groups, like our institution’s Teen Club, to assist in the process. By the last estimate, around 1.8 million adolescents (aged 10–19 years) are living with HIV. This means almost two million young people will be making the transition from being a teenager into young adulthood in the years ahead. We, as a medical community, must work to develop more avenues to assist with this transition and to provide better care, counseling, and support. Right now, Nompilo and other young adults are navigating what it means to grow and thrive as young adults with HIV. When they come in to greet us, we want the answer to “Do you see me?” to be, “Yes!”

Tara Ness and Amanda Small are pediatric residents in the Dr. Kelly DeScioli Global Child Health Residency Program at Baylor College of Medicine. They are currently overseas working at the Baylor College of Medicine - Bristol-Myers Squibb Children’s Clinical Centre of Excellence (COE) in Mbabane, Eswatini, where they will spend a total of 12 months of their residency training.

